# Does the *LHPP* gene share a common biological function in pancancer progression?

**DOI:** 10.1186/s12920-022-01396-5

**Published:** 2022-11-14

**Authors:** Kai Guo, Wei Tian, Hongtao Wang, Dongmin Chang, Yawei Dou, Jinyan Yuan, Yaohua Chen, Bin Hou

**Affiliations:** 1 Department of Thoracic Surgery, Shaanxi Provincial People’s Hospital, Youyi Road 256, Xi’an, 710068 Shaanxi China; 2 Department of Surgical Oncology, The First Affiliated Hospital of Xi’an Jiaotong University, Yanta Road, Xi’an, 710060 Shaanxi China

**Keywords:** LHPP, Tumor suppressor, Pancancer, Bioinformatics

## Abstract

Although emerging evidence has revealed that *LHPP*, a histidine phosphatase protein, suppresses the progression of different cancers, a pan-cancer analysis still remains unavailable. Therefore, we first utilized different bioinformatics tools to explore the tumor inhibitory role of LHPP protein across 33 tumor types based on the TCGA project. Additionally, HGC-27 gastric cancer cells were used to evaluate the biological functions of *LHPP* after stable transfection with lentiviruses. Consequently, *LHPP* mRNA and protein expression were down-regulated in the most cancer tissues corresponding to normal tissues. The data showed that patients with higher *LHPP* performance had a better prognosis of overall survival (OS) and disease-free survival (DFS) in brain glioma and renal carcinoma. In addition, we found that enhancement of *LHPP* expression attenuated the proliferation, migration and invasion of gastric cancer cells. The expression levels of cell-cycle-related and EMT-related molecules, such as CDK4, CyclinD1, Vimentin and Snail, were clearly reduced. Moreover, a genetic alteration analysis showed that the most frequent mutation types in LHPP protein was amplification. The patients without *LHPP* mutation showed a better tendency of prognosis in UCEC, STAD and COAD. Cancer-associated fibroblast infiltration was also observed in head and neck squamous cell carcinoma, stomach adenocarcinoma and testicular germ cell tumors. In summary, our pancancer analysis among various tumor types could provide a comprehensive understanding of *LHPP* biological function in the progression of malignant diseases and promote the development of novel therapeutic targets.

**Supplementary Information:**

The online version contains supplementary material available at 10.1186/s12920-022-01396-5.

## Introduction

Cancer is one of the biggest threats to public health globally. In a recent study [1], 1,898,160 new cancer cases and 608,570 cancer deaths were projected to occur in the United States in 2021. Prostate, bronchus and lung and colorectal cancers (CRCs), which account for 46% of all incident cases, are the three leading cancers in men; breast, bronchus and lung and colorectal cancers, which account for 51%, are three leading cancers in women [1]. Tumorigenesis is an extremely complicated process to study. Therefore, we conducted a pancancer analysis based on the tumor suppressor gene *LHPP,* including its relevance to clinical prognosis and potential molecular mechanisms, to develop new therapeutic targets. Currently, many public databases provide convenience for performing pancancer research, such as TCGA, GEO, and GTEX [2–4]. There have been many pancancer analyses of oncogenes via using these databases.

LHPP (phospholysine phosphohistidine inorganic pyrophosphate phosphatase) protein, also known as HDHD2B, was originally identified in bovine liver and is highly conserved from worms to human beings [5–8]. LHPP protein plays a pivotal role in epigenetics, which is also associated with regulating carcinogenic activity. Recently, LHPP-mediated histidine dephosphorylation was observed in various biological behaviors, such as the development of liver cancer, and the self-renewal of mouse embryonic stem cells and intestinal inflammation. Because of its possible role in histidine phosphorylation, LHPP protein has attracted increasing attention from researchers. In addition, a reduction in *LHPP* expression was observed in different cancer types [5, 9–14]. Our previous study also demonstrated that *LHPP* could repress the proliferation and progression of colorectal cancer cells by inhibiting PI3K/AKT pathway [15]. In addition, our team suggested that LHPP protein might impede the metastasis of colorectal cancer by mediating the phosphorylation of Smad3 in TGF-β/Smad canonical pathway [16]. Based on these results and references, we hypothesized that *LHPP* might be involved in the development of pancancer and play a common role in the progression of different cancer types. Therefore, pancancer analysis was performed to explore potential therapeutic targets by using bioinformatics.

Our research primarily uncovered the expression profile of *LHPP* across 33 cancer types using TCGA, GTEX and GEO databases. In addition to the expression results, we also analyzed the survival status, genetic alteration, immune infiltration of *LHPP* in multiple human cancers. This comprehensive study might provide a new perspective for understanding the progression of tumors.

## Materials and methods

### Gene and protein expression analysis

The Tumor Immune Estimation Resource, version 2 (TIMER2) website [17] (tumor immune estimation resource, version 2, http://timer.cistrome.org/) website provided us with data to analyse the expression difference of *LHPP* between the tumor tissues and adjacent normal tissues.We input *LHPP* into the “Gene_DE” module of TIMER2. Then, the Gene Expression Profiling Interactive Analysis, version 2 (GEPIA2) [18] (http://gepia2.cancer-pku.cn/#analysis) tool was used to obtain box plots of the expression difference of certain tumors with limited normal tissues in TCGA (e.g., TCGA-sarcoma (SARC); TCGA-testicular germ cell tumors (TGCT)) from the Genotype-Tissue Expression (GTEx) database (*P* value cutoff = 0.01; log2FC cutoff = 1). The GTEx [19] data were also used to prove the conclusions obtained from TCGA database. Additionally, the GEPIA2 online tool depicted *LHPP* expression in different pathological stages of various cancers. The log2 [transcripts per million (TPM) + 1] converted expression data were applied to box-line plots or violin plots. Moreover, LHPP protein expression differences between normal tissues and tumor tissues were described via using the UALCAN portal (http://ualcan.path.uab.edu/analysisprot.html) [20, 21] and Clinical Proteomic Tumor Analysis Consortium (CPTAC) dataset [22]. Then immunohistochemistry (IHC) staining images of LHPP expression were downloaded and analyzed from the Human Protein Atlas (HPA) database (http://www.proteinatlas.org/) [23].

### Immunohistochemistry (IHC)

IHC was performed as described previously [15, 16]. Briefly, heterologous tumor tissues and adjacent normal tissues were fixed in formaldehyde and embedded in paraffin. Before immunostaining, 4-µm-thick tissue sections were dewaxed in xylene and washed three times in PBS. Then, goat serum (10%) was used to block nonspecific staining for approximately 30 min. Sections were incubated with rabbit polyclonal antibodies against LHPP (dilution 1:200; Catalog no. 15759-1-AP; Proteintech) overnight at 4 °C. Two independent investigators evaluated staining blindly.

### Western blot assay

Western blot analysis was performed as described previously [15, 16]. Briefly, total protein was extracted from cells or tissues using RIPA buffer with protease inhibitors. Equal amounts of protein (20–30 µg) were separated via SDS-PAGE (10–12% gel) and then transferred to PVDF membranes. The membranes were blocked with 5% milk and incubated with primary antibodies overnight at 4 °C, followed by incubation with the secondary antibody (dilution 1:5000) at room temperature. The bands were visualized using Immobilon Western Chemilum HRP Substrate (cat. no. WBKLS0100; Millipore).

### Survival prognosis analysis

Subsequently, we focused on how *LHPP* expression correlated with prognosis including overall survival (OS) and disease-free survival (DFS). Significance map data and survival plots of *LHPP* across all TCGA tumors were described using the GEPIA2 module. All cases were divided into high-performance and low-performance queues with cutoff values of high (50%) and low (50%). The log-rank test was used to evaluate significant differences between two groups.

### Genetic alternation analysis

The genetic alteration data, including alternation frequency, mutation type, mutated site, copy number alternation (CNA), and three-dimensional structure of LHPP protein, were downloaded from the cBioPortal (CBP) tool (https://www.cbioportal.org/) [24]. To obtain overall, disease-free, progression-free survival data, the “Comparison” module in CBP was used to analyze significant differences with or without *LHPP* genetic alternation. Kaplan‒Meier plots with log-rank P values are depicted as well.

### The relationship between *LHPP* expression and the tumor microenvironment

The correlation between *LHPP* expression and the tumor micro-environment was explored by using the ‘Immune-Gene’ module of the TIMER2 online tool. We selected cancer-associated fibroblasts, CD8 + T cells and endothelial cells for further analysis. The TIMER, CIBERSORT, CIBERSORT-ABS, QUANTISEQ, XCELL, MCPCOUNTER and EPIC algorithms were used for immune infiltration estimations [25, 26]. The *P* values and partial correlation (cor) values were calculated by purity-adjusted Spearman’s rank correlation test. The heatmaps and scatter plots are shown.

### Cell culture

The human gastric cell line HGC27 was purchased from the Cell Bank of the Chinese Academy of Sciences (*Shanghai China*) and cultured in complete RPMI 1640 medium (*HyClone, USA*). The medium was supplemented with 10% fetal bovine serum (*FBS Gibco*) and a 1% penicillin–streptomycin mixture. Cells were maintained at 37 °C in a humidified incubator with 5% CO_2_.

### Cell transfection

The cell transfection protocol was described previously [15, 16]. A total of 20–30% of cells were stably transfected with *LHPP* lentiviruses (LV) or negative control LVs (NC) according to the manufacturer’s protocol (*viral volume* = *MOI* × *cell numbers/viral titers; GeneChem Co., Ltd, Shanghai, China; MOI* = *20, cell numbers: 1*–*5* × *10*^*5*^*, viral titers: 4* × *10*^*8*^). Lentiviral vectors used the GV358 system combined with the cytomegalovirus promoter-driven puromycin gene and green fluorescent protein.

### Colony formation assay

The colony formation assay was described previously [15, 16]. Briefly, cells were plated (1000–2000 per well) in culture plates for 3–4 weeks at 37 °C in a humidified environment and then stained with crystal violet staining solution (1%) for 30 min. The stained colonies were imaged using a camera and counted using a microscope. Only colonies containing more than 50 cells were counted.

### Transwell assays

The Transwell assays were described previously [15, 16]. Briefly, Transwell cells (1 × 105) were plated on top of a 24-well Corning 8 μm pore membrane with serum-free medium. The matrix gel was added on top of the Transwell chamber (BD, USA, dilution 1:8, 50–80 μl, diluted with medium). After 24–48 h, invading cells were fixed with 4% paraformaldehyde and stained with crystal violet. Microscopy was used to image migrating cells and count cell numbers.

### Statistical analysis

The statistical data were calculated using GraphPad Prism 6 software. A two-tailed Student’s t test was used to analyze the statistical significance between different groups. *P* < 0.05 was regarded as statistically significant.

## Results

### *LHPP* gene expression in different tumors

**Fig. 1:**
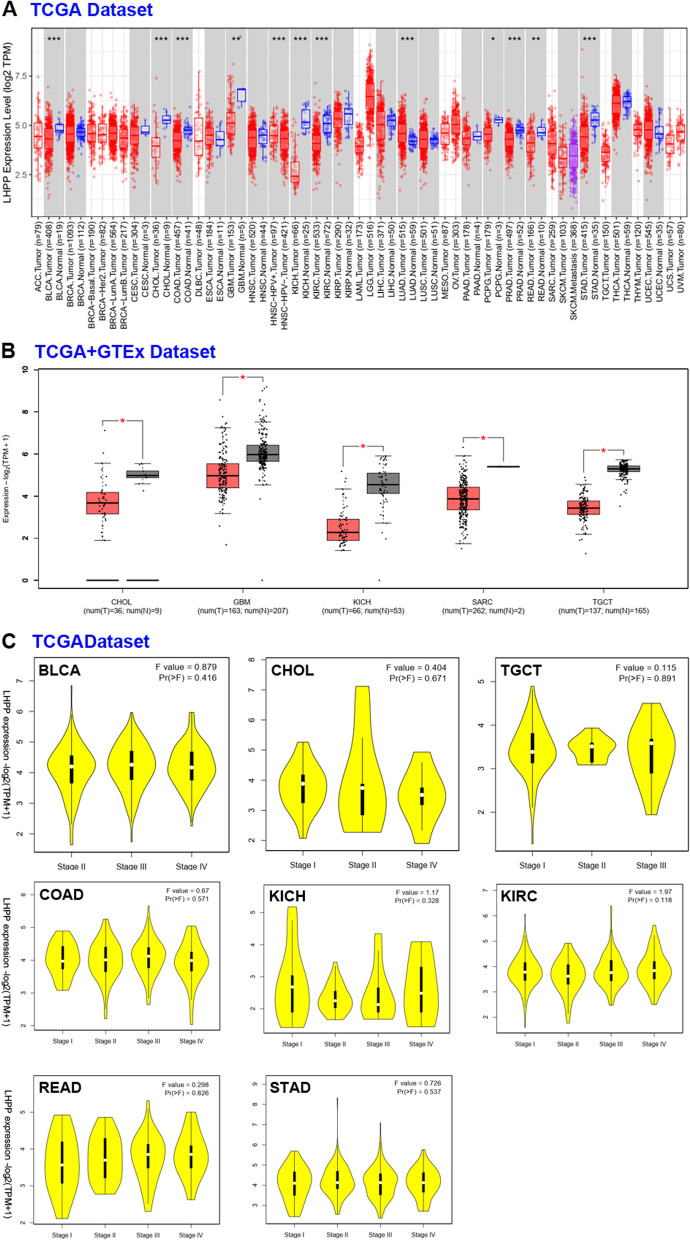
mRNA expression level of LHPP in different human cancer types. **A** mRNA expression level of LHPP in TCGA tumor tissues compared to adjacent normal tissues as available by TIMER2. *P < 0.05, **P < 0.01, ***P < 0.001; **B** Box plot of LHPP expression level comparison in CHOL, GBM, KICH, SARC and TGCT (TCGA project) relative to the corresponding normal tissues (GTEx database). *P < 0.05; **C** Results of LHPP gene expression levels in different tumor pathological stages

Primarily, we evaluated the expression levels of the *LHPP* gene among all 33 cancer types in the TCGA database. Consequently, 11 kinds of cancers were significantly changed, accounting for 33.3% of all types. The *LHPP* expression level was apparently higher in normal tissues of bladder urothelial carcinoma (BLCA), cholangiocarcinoma (CHOL), colon adenocarcinoma (COAD), glioblastoma multiforme (GBM), kidney chromophobe (KICH), kidney renal clear cell carcinoma (KIRC), pheochromocytoma and paraganglioma (PCPG), prostate adenocarcinoma (PRAD), and stomach adenocarcinoma (STAD) than in the corresponding cancer tissues (Fig. 1A). In contrast, the *LHPP* gene had obviously lower expression in lung adenocarcinoma (LUAD) normal tissues than in adjacent cancer tissues (Fig. 1A).

For tumors without normal tissues matched as controls, such as lymphoid neoplasm diffuse large B-cell lymphoma (DLBC), acute myeloid leukemia (LAML), brain lower grade glioma (LGG), ovarian cystadenocarcinoma (OV), sarcoma (SARC), testicular germ cell tumors (TGCT), thymoma (THYM) and uterine carcinosarcoma (UCS), we further examined the *LHPP* expression data in GTEx. As shown in Fig. 1B, *LHPP* gene expression levels were definitely reduced in tumor tissues compared with normal tissues among sarcoma (SARC) and testicular germ cell tumors (TGCT). Additionally, we did not find a difference in LGG or UCS. However, *LHPP* expression in cancer tissues was apparently increased in DLBC, LAML, OV and THYM. Generally, we found that *LHPP* expression levels were impaired in the majority of human tumors, which suggested that the *LHPP* gene might serve as a tumor suppressor. In addition, we utilized the GEPIA2 online tool to reveal the relationship between *LHPP* expression and tumor pathological stages. In most tumor types, there was no clear association between *LHPP* expression and pathological stage (Fig. 1C).

**Fig. 2:**
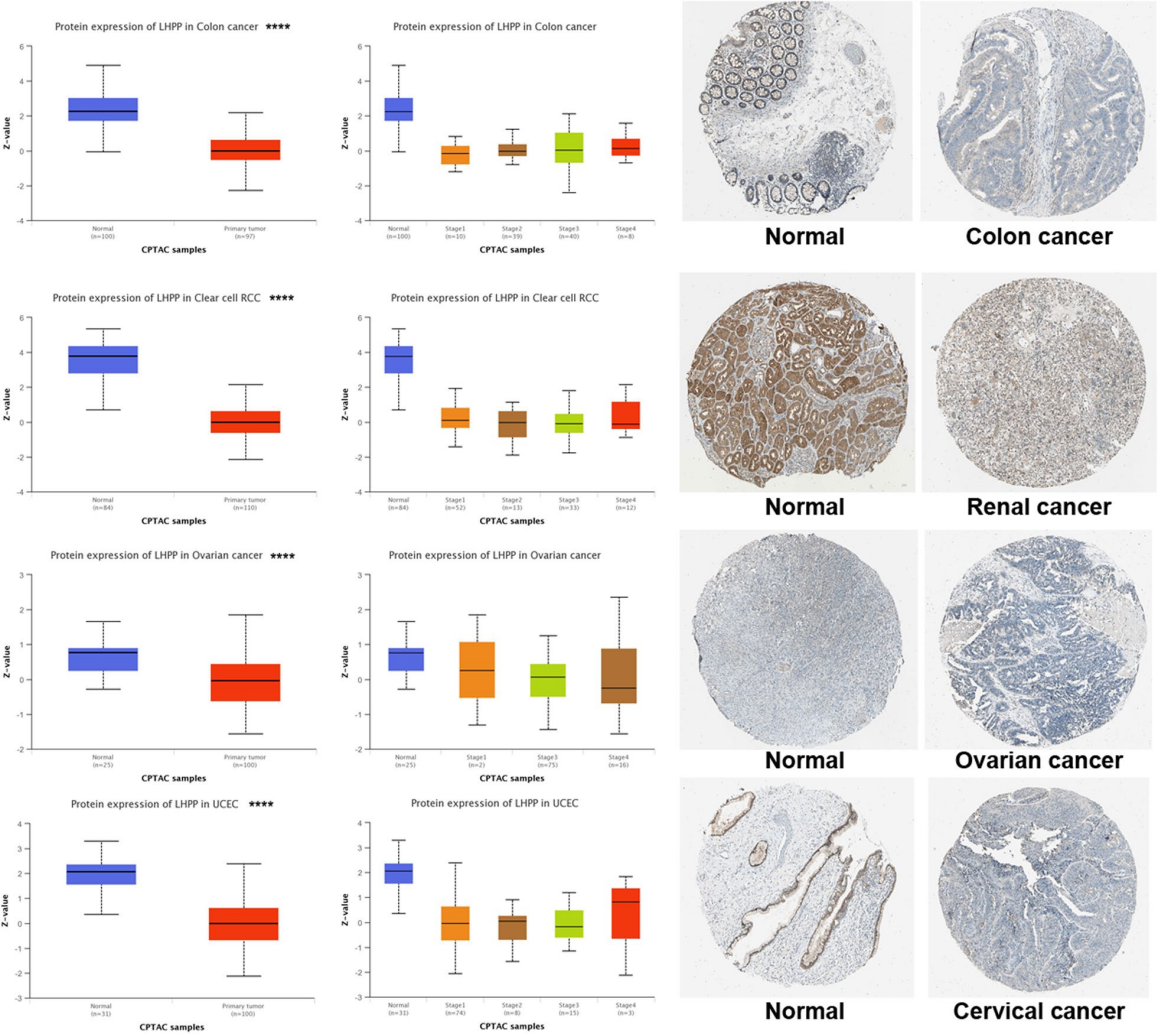
LHPP protein expression levels in normal tissues and primary ovarian cancer, clear cell RCC, colon cancer and UCEC were extracted and analyzed using CPTAC; all *P* values were less than 0.001. Typical images of IHC were downloaded from the HPA database

In addition to transcription, LHPP protein levels were also assessed using the National Cancer Institute’s CPTAC dataset. The data suggested that the total protein level of LHPP was remarkably lower in COAD, RCC, OV and UCEC cancer tissues than in the corresponding controls (Fig. 2, all *P* < *0.001*). Interestingly, we found that LHPP protein levels were obviously attenuated at the initial stage of tumor development, which suggested that LHPP protein might play an important role in maintaining the development of normal tissues. Moreover, we downloaded representative images of IHC from the HPA database. The results were consistent with previous conclusions (Fig. 2).

### Survival data analysis

**Fig. 3:**
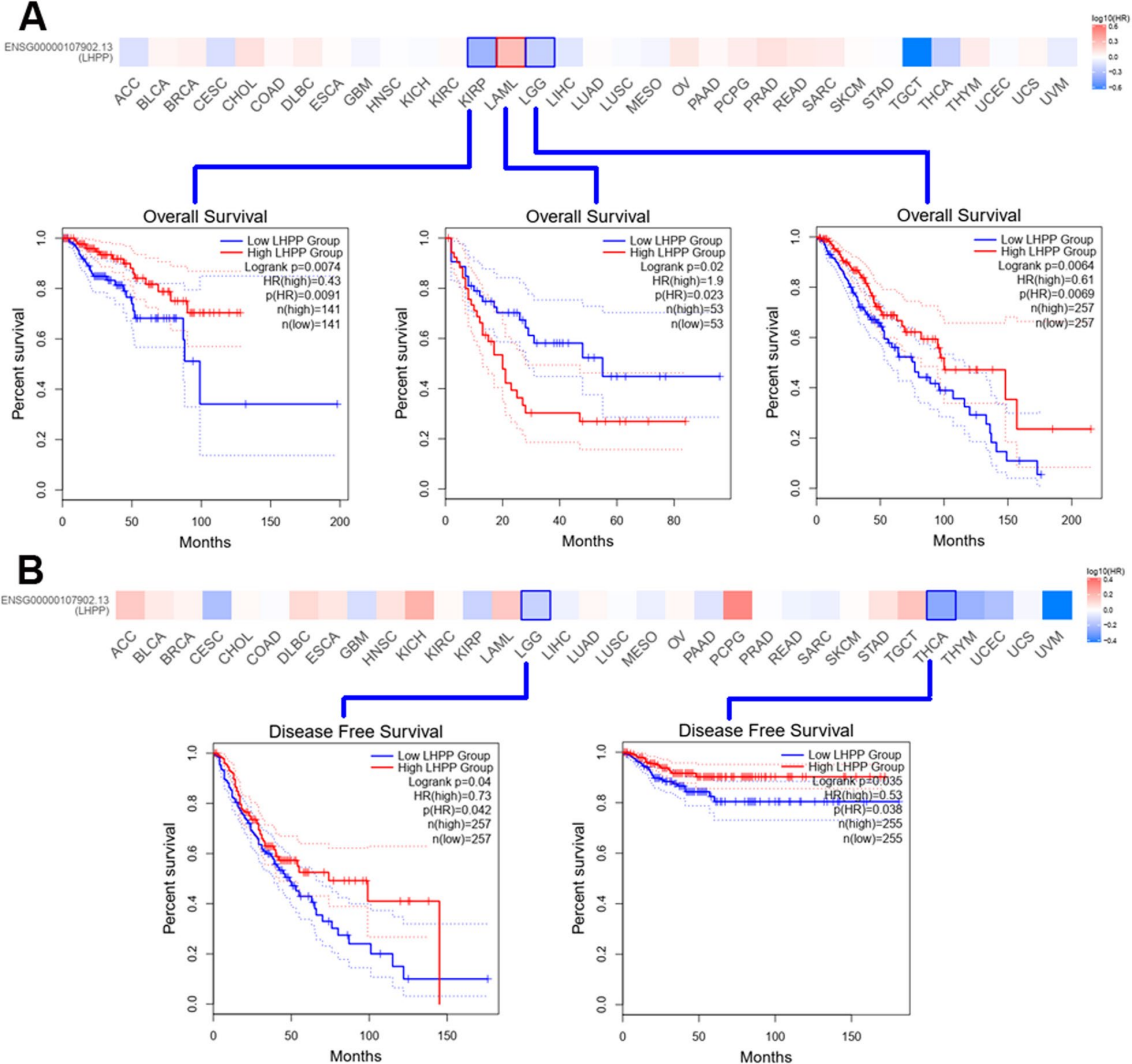
Association between LHPP gene expression and patient clinical prognosis from the TCGA dataset. Overall survival (**A**) and disease-free survival (**B**) were evaluated by using Kaplan‒Meier curves

Next, taking advantage of the TCGA and GEO databases, we paid attention to the correlation between *LHPP* gene expression and patient prognosis, including overall survival (OS) and disease-free survival (DFS). Cancer cases were divided into two groups according to the performance status of *LHPP* gene expression, namely, the high-expression group and the low-expression group. As presented in Fig. 3A, high expression of the *LHPP* gene was closely associated with better prognosis of overall survival in KIRP (*P* value = 0.0074) and LGG (*P* value = 0.0064) in the TCGA project. Disease-free survival data (Fig. 3B) also elucidated a similar tendency that a better prognosis could be observed in LGG (*P* value = 0.04) and THCA (*P* value = 0.035). Conversely, we found that high *LHPP* expression was markedly connected with a poorer prognosis of OS in LAML (*P* value < 0.02). Overall, as we expected, patients with high *LHPP* expression had a better prognosis of OS and DFS, which indicated that *LHPP* might suppress the progression of tumors.

### Genetic alteration data

**Fig. 4:**
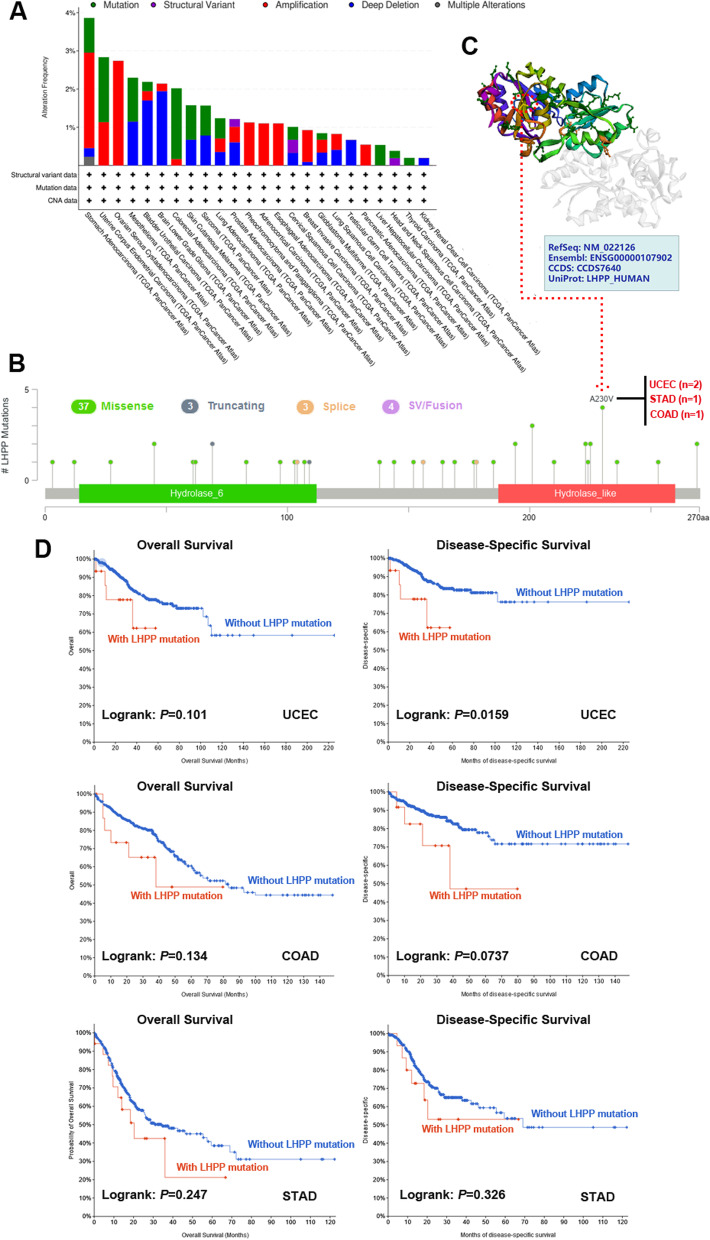
Genetic alteration status of LHPP was presented by using the cBioPortal tool. **A** The alteration frequency of LHPP with various mutation types is displayed. **B** Mutation sites of LHPP were also analyzed by utilizing the cBioPortal tool. **C** Analysis of the relationship between genetic mutations and overall survival (OS) and disease-specific survival (DSS) in patients with UCEC, COAD and STAD

Generally, the accumulation of genetic alterations causes the development of human cancers. Therefore, in this study, the genetic pattern of the LHPP gene was further analyzed in different cancer types from the TCGA project. There were four main genetic alterations: mutation, structural variant, amplification, and deep deletion. According to our data (presented in Fig. 4A), amplification was the most common genetic alteration type, mainly occurring in stomach adenocarcinoma, uterine corpus endometrial carcinoma, ovarian serous cystadenocarcinoma, adrenocortical carcinoma, esophageal carcinoma, pheochromocytoma and paraganglioma. Additionally, the highest frequency of *LHPP* genetic alteration (3.86%) was occurred in stomach adenocarcinoma with amplification as the primary alteration type, and the rate of amplification in STAD was 2.50%. Among different cancer types, LGG possessed the highest occurrence of the ‘deep deletion’ type, with a frequency of 1.95%. Interestingly, we noticed that brain lower grade glioma patients (LGG) with high LHPP expression had better overall survival and disease-free survival (Fig. 3). Therefore, we further analyzed *LHPP* genetic alterations in LGG patients. The results showed that missense mutations were the main genetic alternation in LHPP, which was consistent with our findings in subsequent research research (Fig. 4). The R45H* mutation in the Hydrolase domain has been studied in 1 case of LGG, which might induce a translation from R (arginine) to H (histidine) at the site 45 of LHPP protein (Additional file 1: Fig. S1). The 3D structure of the LHPP protein with R45H site is presented in Additional file 1: Fig. S1B. In addition, the highest incidence of ‘mutation’ was found in colorectal cancer, with 1.85% frequency. Moreover, the types, sites and number of cases of *LHPP* genetic changes are shown in Fig. 4B. Detailed analysis uncovered that missense mutation was the main genetic alteration in LHPP. For example, the A230V* mutation in the Hydrolase_like domain has been studied in 2 cases of UCEC, 1 case of STAD and 1 case of COAD, which might induce a translation from A (alanine) to V (valine) at the site 230 of the LHPP protein. The 3D structure of the LHPP protein with A230V site is presented in Fig. 4C. We systematically investigated the relationship between *LHPP* gene mutation and clinical prognosis in various cancer types. The clinical results in Fig. 4D show that UCEC patients without *LHPP* gene changes presented a better disease-specific survival (*P* values = 0.0159) than patients with *LHPP* gene mutations, but not in OS (*P* values = 0.101). Although, a significant difference could not be observed between patients with *LHPP* gene mutations and those without gene mutations, we found COAD and STAD patients without LHPP gene changes tended to have better clinical prognoses.

### The biological function of *LHPP* in STAD cancer

**Fig. 5:**
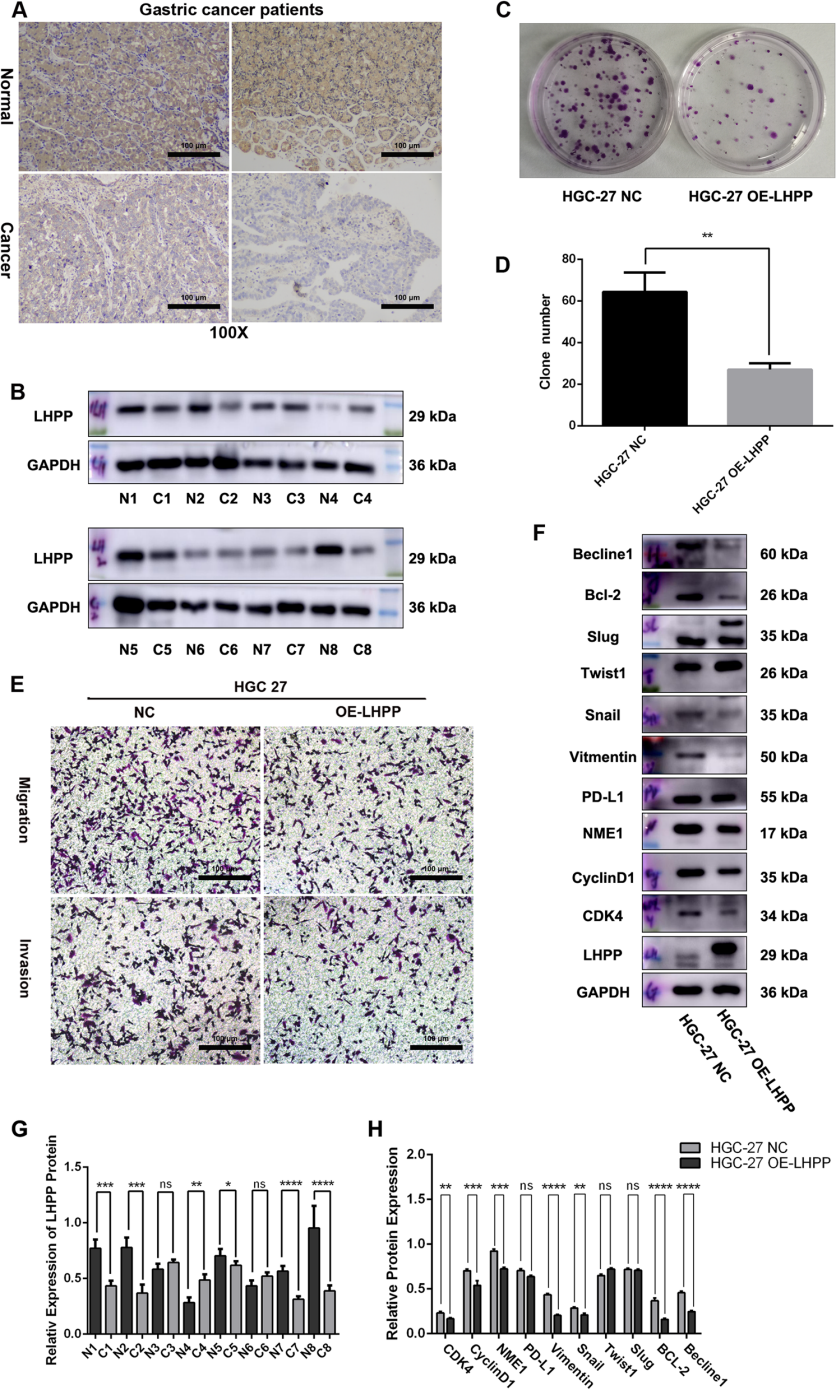
The biological functions of the LHPP gene were determined in gastric cancer. **A** LHPP protein expression level was detected via IHC in cancer tissues vs. normal tissues. **B** LHPP protein expression was downregulated in cancer tissues compared to their corresponding normal tissues. **C** and **D** The effect of LHPP upregulation on the proliferation of HGC-27 cells was determined via a colony formation assay. A significant difference was observed by using GraphPad Prism 6 software. (E) A Transwell assay was utilized to examine the migration and invasion (matrix gel 50–80 μl, dilution 1:8, 8-μm pore) abilities of HGC-27 cells after overexpressing LHPP protein. **F** The cell cycle-related and EMT-related biomarkers, such as CDK4, CyclinD1, NME1, BCL-2, Vimentin, Snail, Slug and Twist1, were evaluated by using a Western blot assay. **G** The relative expression of LHPP protein was examined between gastric cancer tissues and matched normal tissues via ImageJ and GraphPad Prism 6 software. **H** The relative expression levels of related proteins were calculated using ImageJ and GraphPad Prism 6 software. *P < 0.05, **P < 0.01, ***P < 0.001, ****P < 0.0001

As we have studied the function of *LHPP* in colorectal cancer and esophageal cancer [15, 16], we selected the gastric cancer cell line HGC-27 for subsequent research. We performed Western blot assays and immunohistochemistry to explore *LHPP* expression in normal tissues and cancer tissues (n = 20). Consistently, our results demonstrated that *LHPP* expression in normal tissues was definitely higher than that in cancer tissues (Fig. 5A, B). In addition, HGC-27 cells with low expression of the *LHPP* gene were stably transfected with *LHPP* overexpression lentiviruses (*OE-LHPP*) or corresponding negative control lentiviruses (*NC*). As presented in Fig. 5C and D, the colony formation assay indicated that *LHPP* enhancement could significantly repressed HGC-27 cell proliferation (HGC-27 *NC vs.* HGC-27 *OE-LHPP P* < 0.01).In addition, the Transwell assay also suggested that *LHPP* overexpression might attenuate the migration and invasion abilities of HGC-27 cells (Fig. 5E). The cell number in the HGC-27 *OE-LHPP* groups was obviously lower than cells in negative control groups. As we expected, the key molecules of the cell cycle and EMT-related proteins, such as CDK4, CyclinD1, Vimentin, Snail and Bcl-2, were markedly reduced after *LHPP* gene overexpression (Fig. 5F, H). Interestingly, we found that a bio-marker of cell autophagy, Beclin1, was also suppressed in the *LHPP* over-expression group.

### Immune infiltration analysis results

**Fig. 6:**
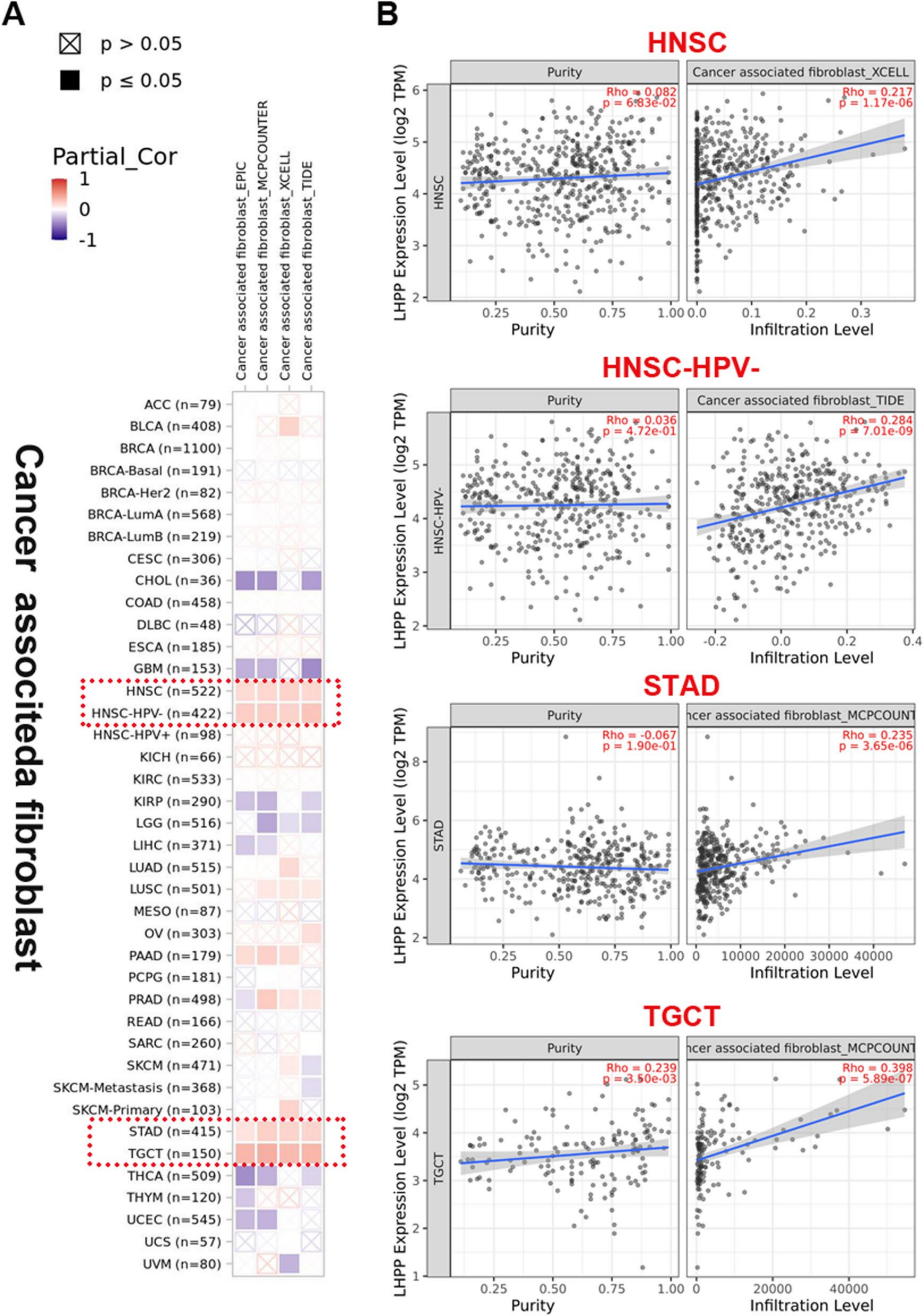
TIMER2 was used to explore the relationship between LHPP expression and immune infiltration of cancer-associated fibroblasts. **A** EPIC, XCELL, MCPCOUNTER and TIDE algorithms were used to study the correlation between the expression level of the LHPP gene and the infiltration level of cancer-associated fibroblasts. **B** The relationship of LHPP and the infiltration level of cancer-associated fibroblasts across HNSC, HNSC-HPV(-), STAD, and TGCT

Recently, increasing evidence has suggested that cancer-associated fibroblasts are involved in the development and distant metastasis of various cancer types by modulating the functions of different immune cells. Therefore, we investigated the potential correlation between *LHPP* gene expression and the infiltration level of tumor fibroblasts in multiple cancer types from the TCGA project by using the TIMER, CIBERSORT, CIBERSORT-ABS, TIDE, XCELL, MCPCOUNTER, QUANTISEQ and EPIC algorithms. Interestingly, we discovered that *LHPP* gene expression was positively associated with the infiltration value of cancer-associated fibroblasts in HNSC, STAD and TGCT. Conversely (Fig. 6A), a statistically negative correlation between *LHPP* performance and cancer-associated fibroblasts was observed in CHOL, GBM, LGG and THCA cancers (Additional file 2: Fig. S2). Additionally, the scatter plot data obtained by the algorithm for the above tumors are illustrated in Fig. 6B and Additional file 2: Fig. S2. For instance, according to the XCELL algorithm, LHPP performance in HNSC had a positive correlation with cancer-associated fibroblasts (Cor = 0.217, *P* value = 1.77e−06), and LHPP expression levels in LGG were negatively associated with cancer-associated fibroblasts according to the MCPCOUNTE algorithm (Cor = − 0.33, *P* value = 1.28e−13).

## Discussion

*LHPP,* a kind of histidine phosphatase protein, is a transmembrane-free protein mainly located in the cytoplasm. *LHPP* is closely associated with protein homodimerization and inorganic diphosphatase activity [5, 6, 8]. Recently, an increasing number of publications have suggested that *LHPP* serves as a tumor suppressor in different tumor types, such as hepatocellular carcinoma (HCC) [5, 27], intrahepatic cholangiocarcinoma (ICC) [11], glioblastoma [28], melanoma [29], cervical [14], bladder [10], thyroid [30], pancreatic [12], renal [13] and colorectal cancers [15, 16]. A reduction in *LHPP* expression is highly correlated with poor prognosis in patients with malignant diseases. Mechanistically, *LHPP* suppresses cancer cell proliferation, metastasis, and energy metabolism and promote cell apoptosis and autophagy. Interestingly, Xia et al. illustrated [31] that *LHPP*-mediated histidine dephosphorylation repressed self-renewal of mouse embryonic stem cells by mediating the mRNA or protein expression of β-catenin, CDK4 and CyclinD1. Therefore, we chose to explore *LHPP* functions systematically, in addition to other molecular features and genetic alterations.

In this research, a lower expression level of *LHPP* was observed in most cancer tissues than in their corresponding normal tissues, such as BLCA, CHOL, COAD, GBM, KICH, KIRC, PCPG, PRAD, and STAD. Consistently, our previous study [15] revealed that patients with higher *LHPP* expression exhibited smaller tumor size, lower TNM stage and better prognosis. In addition, we also evaluated *LHPP* expression in STAD and ESCA tissues. As expected, *LHPP* expression was clearly higher in normal STAD tissues than in cancer tissues (Fig. 5A, C). In addition, clone formation assay and Transwell assay demonstrated that increasing *LHPP* expression repressed HCG-27 cell growth, proliferation and metastasis (Fig. 5). Biomarkers of the cell cycle and EMT, such as CDK4, CyclinD1, BCL-2, Snail, and Vimentin, were also reduced in the HCG-27 *LHPP*-overexpression group. However, we did not find statistical significance in patients with esophageal carcinoma. Interestingly, mRNA expression differences were not observed in OV or UCEC, while LHPP protein expression was markedly decreased in OV and UCEC cancer tissues. This result might suggest that the regulation of LHPP posttranscription was existed in the development of OV and UCEC cancers. Furthermore, high *LHPP* performance was observed in LUAD cancer tissues. Conversely, Yang et al. suggested that *LHPP* mRNA expression was reduced in NSCLC tissues, and the miR-217/LHPP [32] axis was also activated in the reduction of cisplatin resistance in NSCLC.

The relationship between patient prognosis and *LHPP* expression performance was also investigated in this paper. We made several meaningful conclusions in our analysis. Especially in brain glioma, the data demonstrated that patients with high *LHPP* expression have a better prognosis of overall and disease-free survival. The reason might be explained by Chen. His research [28] proved that *LHPP* could regulate the cancer energy metabolic process via impeding glycolysis and respiration through the induction of ubiquitin-mediated degradation of PKM2 in glioblastoma. Although the clinical prognosis of most cancer types was not correlated with *LHPP* expression levels after bioinformatics analysis, the clinical data from other references, including data on BLCA [10], LIHC [5, 33], PAAD [12], CHOL [11] and COAD [15] provide markedly positive pathological maps with high *LHPP* performance. This mounting evidence strongly provs that LHPP protein might be an inhibitor of malignant cell transformation. At this point, upregulation of *LHPP* expression might be an effective way to improve the overall survival of patients with malignant diseases.

An increasing number of studies have explained the molecular mechanisms of *LHPP* biological functions in the development of various tumor types, but the mutational features of LHPP protein still remain unknown. We analyzed the correlation between LHPP genetic alterations and patient prognosis. The most frequent mutation type in *LHPP* was amplification, which was mainly observed in ovarian serous cystadenocarcinoma. In addition, missense mutations were the most common changes in *LHPP,* which always occurred in the hydrolase-like domain. Especially, translation from (alanine) to V (valine) at site 230 was the typical change in LHPP protein (but not in LGG). After Kaplan‒Meier analysis, we found that patients without *LHPP* gene mutation had a tendency toward better clinical prognosis in UCEC, COAD and STAD. These results were consistent with LHPP expression data in the TCGA project. Furthermore, the expression of the LHPP gene was suppressed in STAD and COAD cancer tissues. These data suggest that missense mutations were associated with downregulation of LHPP gene expression. Recent research in prostate cancer revealed that YTHDF2 induced LHPP gene alteration by directly binding to m6A-modified sites of LHPP, which supports our above findings. In addition, LGG patients with high LHPP expression had better overall and disease-free survival. We supposed that LHPP gene missense mutations were also associated with poor clinical prognosis. Surprisingly, there was no significant difference between UCEC cancer tissues and normal tissues, which warrants further research.

Mechanistically, as a kind of histidine phosphatase with homodimerization and diphosphatase activity, the LHPP protein is mainly correlated with substance metabolism, which has been proved in colitis, hepatocellular carcinoma [5] and embryonic stem cells [31]. Additionally, the dephosphorylation function of LHPP protein has been also widely studied in multiple cancers. *LHPP* deregulated AKT and mTOR phosphorylation to repress cell proliferation and progression in colorectal [15], cervical [14], bladder [10] and thyroid [30] cancers. Our previous study [16] and a study by Dan Wang et al. [11] also found that *LHPP* inhibited the migration and invasion abilities of colorectal cancer and ICC by mediating Smad2/Smad3 phosphorylation in the TGF-beta pathway. Therefore, we supposed that *LHPP* might serve as a tumor suppressor by downregulating the phosphorylation of oncogenes during the progression of most cancers.

In summary, according to our systematic pancancer analysis of the LHPP gene, we found a correlation between LHPP expression and clinical prognosis, immune cell infiltration, and tumor mutation burden for a variety of human cancers. These conclusions suggested that LHPP might be a tumor inhibitor for developing novel therapeutic targets. Nevertheless, there were some limitations in this research. First, a greater number of pathological specimens should be collected to examine LHPP expression between normal and cancer tissues. A clinical study is also important for future research. Second, other cell lines from different cancer types should be used to define the biological functions of LHPP protein. Finally, this research lacked a detailed clarification of the molecular mechanisms of LHPP. These problems deserve further research.

## Supplementary Information

**Additional file 1: Fig. S1.** Genetic alteration status of LHPP was presented in brain lower grade glioma by using the cBioPortal tool. **A** The alteration frequency of LHPP with various mutation types is displayed. **B** Mutation sites of LHPP were also analyzed by utilizing the cBioPortal tool.

**Additional file 2: Fig. S2.** TIMER2 was used to explore the relationship between LHPP expression and immune infiltration of cancer-associated fibroblasts. **A** EPIC, XCELL, MCPCOUNTER and TIDE algorithms were used to study the correlation between the expression level of the LHPP gene and the infiltration level of cancer-associated fibroblasts. **B** The relationship of LHPP and the infiltration level of cancer-associated fibroblasts across CHOL, GBM, LGG, and THCA.

## Data Availability

The data that support the findings of this study are available from the corresponding author upon reasonable request and online database.
