# Supplementary figures and images for: Does the LHPP gene share a common biological function in pancancer progression?
Source: BMC Med Genomics. 2022 Nov 14;15:239. doi: 10.1186/s12920-022-01396-5 (PMC9661738; doi:10.1186/s12920-022-01396-5)

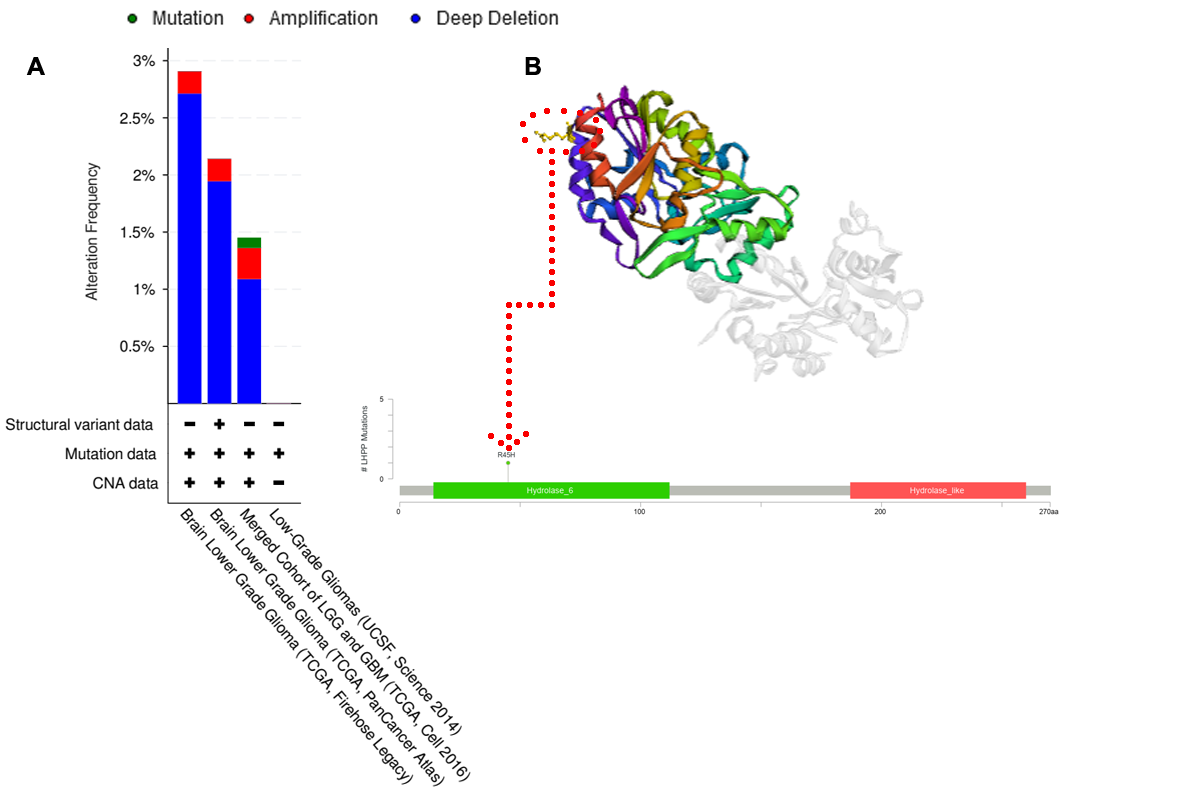

Supplement: Supplementary file 1 — Additional file 1: Fig. S1. Genetic alteration status of LHPP was presented in brain lower grade glioma by using the cBioPortal tool. A The alteration frequency of LHPP with various mutation types is displayed. B Mutation sites of LHPP were also analyzed by utilizing the cBioPortal tool. [file 12920_2022_1396_MOESM1_ESM.tif]

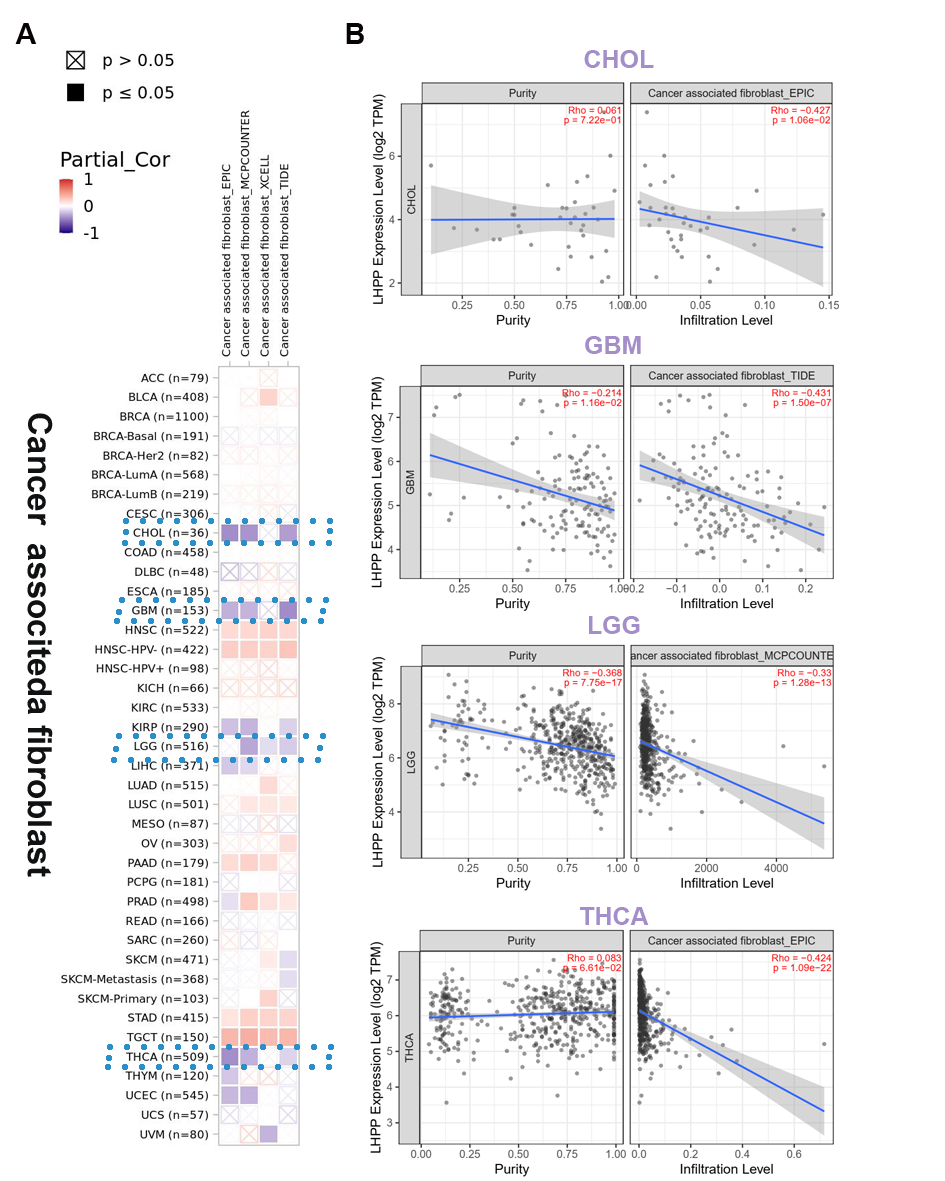

Supplement: Supplementary file 2 — Additional file 2: Fig. S2. TIMER2 was used to explore the relationship between LHPP expression and immune infiltration of cancer-associated fibroblasts. A EPIC, XCELL, MCPCOUNTER and TIDE algorithms were used to study the correlation between the expression level of the LHPP gene and the infiltration level of cancer-associated fibroblasts. B The relationship of LHPP and the infiltration level of cancer-associated fibroblasts across CHOL, GBM, LGG, and THCA. [file 12920_2022_1396_MOESM2_ESM.tif]
